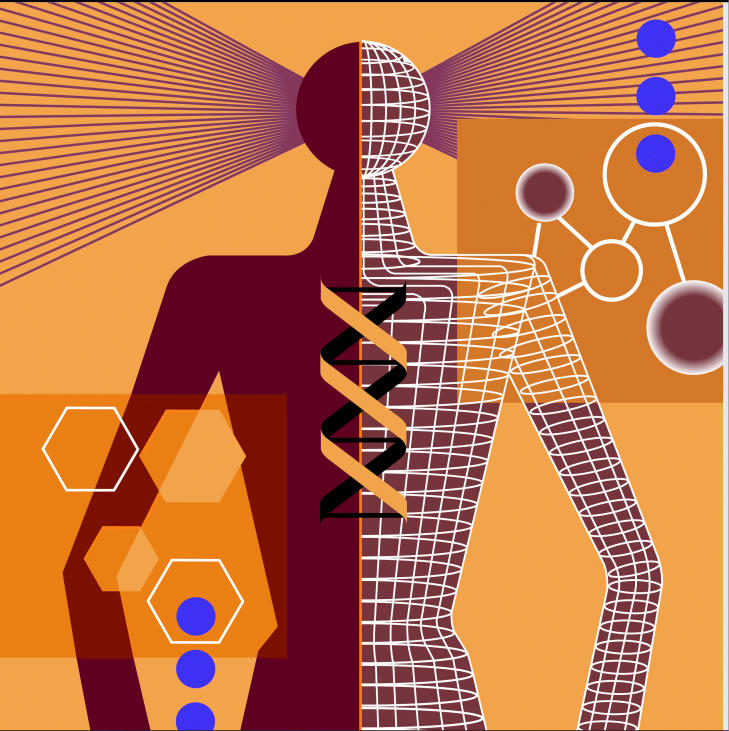# Systems Biology: The Big Picture

**DOI:** 10.1289/ehp.112-a938

**Published:** 2004-11

**Authors:** Angela Spivey

Genomics, proteomics, and metabolomics have all vastly advanced our understanding of human biology and disease. But the functioning of even a simple system such as a single yeast cell or bacterium is much more complicated than the sum of its genes or proteins or metabolites; it’s the activity of all those components and their relationships to one another that add up to a living organism. Recognizing that complexity, the emerging field of systems biology attempts to harness the power of mathematics, engineering, and computer science to analyze and integrate data from all the “omics” and ultimately create working models of entire biological systems.

“Traditionally, scientists—toxicologists included—have relied on a reductionist approach to biology,” says William Suk, director of the NIEHS Center for Risk and Integrated Sciences. Even now, many studies examine complex systems by looking at cellular components in isolation. For instance, a common experiment involves using DNA microarrays to observe the effect of a chemical exposure on thousands of genes at once. This technique can quickly tell a scientist which genes may be vulnerable to that exposure. But a systems biology approach would attempt to model not only the chemical’s effect on gene expression but also how that expression will affect protein function, and in turn how the exposure will affect cell signaling. “There’s nothing wrong with what we’ve been doing,” Suk says. “But systems biology is going to take it to another level.”

## Building a New Science

From one perspective, systems biology is nothing new. At the turn of the twentieth century, physiologists such as Walter B. Cannon were developing the concept of homeostasis—the self-regulatory mechanisms, hunger and thirst for example, that a living organism uses to keep its internal systems in balance despite an ever-changing external environment. The term “systems biology” was first used in the 1960s, when theoretical biologists began creating computer-run mathematical models of biological systems.

We’re exposed to lots of chemicals but at very low concentrations over time. We need tools to help us understand how complex exposures perturb complex systems.

— William Suk

But the field took a leap forward beginning in the 1990s, when the high-throughput tools developed for the sequencing of the human genome brought experimental scientists up to the speed of theoretical biologists. The widespread use of the Internet has also made possible for the first time the international collaborations and sharing of huge amounts of data that systems biology requires. “The way that computer science has responded to genomics is one of the great stories of the sociology of twentieth-century science,” says Charles DeLisi, senior associate provost for bioscience and chair of the Bioinformatics Program at Boston University. Computer scientists have taken a great interest in biology and have stepped up to collaborate with biologists to develop the tools needed to sequence genomes and analyze the resulting data.

Leroy Hood, a biochemist who is president and cofounder of the nonprofit Institute for Systems Biology, agrees. “What uniquely defines the systems biology that I’m thinking about has really come from the genome project and its delineation of a complete parts list of all the genes,” he says. “If you know all the genes, you have the ability to do DNA arrays, follow the behavior of all the messenger RNAs, and even the proteins, in principle.”

Measuring gene expression is one important component of systems biology, and methods for doing so are fairly well developed for the needs of this field. However, proteomics—the science of analyzing all the proteins present in a system at any one time—still has some maturing to do before scientists can integrate its data payload into a true systems approach. Proteomics has been hailed as having even more potential than genomics, because whereas DNA is a set of static instructions for an organism, proteins—the machines that actually carry out the work—are a more fluid medium and may reflect the effects of chemical exposure more accurately. But to integrate protein expression into systems biology, scientists need to better understand its relationship to gene expression. DeLisi says scientists still don’t understand why in many cases there is a tight correlation between gene and protein expression, while in others (as with transcription factors) the correlation is very loose. “That [understanding] will develop over the next five to ten years,” he predicts.

Other researchers have pointed to the need for more quantitative techniques to not only detect the presence of proteins but also determine their size, purity, and concentration in a system. Hood agrees that to achieve truly global analyses of complex biological systems, proteomics technology needs more development. “The big problem that proteomics has is that proteins that are expressed at very low levels are generally invisible to the analytic techniques we have,” he says. Hood suggests the answer to that problem lies in highly miniaturized sensors and detectors developed through nanotechnology and microfluidics.

Another “omics” that promises to fill in gaps in systems biology models is metabolomics—the evaluation of tissues and biofluids such as urine, blood plasma, and saliva for metabolite changes that may result from environmental exposures or from disease. Because metabolites (which include carbohydrates, amino acids, and lipids) are the actual by-products of processing food into energy, this “omics” has the potential to paint a picture of what has actually happened in the cell.

Work in metabolomics seeks to go beyond sampling single metabolites to developing profiles of four or five related metabolites. But to create meaningful metabolite profiles, scientists need better tools for measuring tiny amounts of metabolites and for determining which are most important in the activity of the cell. Existing analytical tools such as nuclear magnetic resonance spectrometry and mass spectrometry go only so far. Indeed, the Metabolomics Technology Development initiative of the NIH Roadmap for Medical Research encourages projects that develop new methods for measuring metabolites that are present only in low concentrations or at specific subcellular locations.

## The Measure Is in the Models

For those working on a systems biology approach, the goal of developing these various “omics” technologies is to combine their data into interactive models. Hood says, “The ultimate object of systems biology is to understand how the elements and their interactions together give rise to the emergent properties of the system.”

To do that, scientists begin by modeling individual components such as protein networks and signal transduction pathways. Initially, says Hood, the models are descriptive. They may involve perhaps a relatively simple equation showing relationships between a few proteins in a cell. As more information comes to light, the models become more graphical. A graphical model may visualize a cell as a very complicated flow chart, as a series of pinwheels, or as a spiderweb. Relationships between elements are depicted through color or distance.

Next, researchers can experiment with an actual system such as yeast to see what will happen at the organism level when one component of the system is perturbed. “You can do genetic perturbations where you knock out genes, for example, or environmental perturbations where you give or take away certain kind of sugars,” Hood says. “And then you observe how all the other elements behave in response to those perturbations.” Those experiments will usually yield data that aren’t explained by the model. “So you formulate hypotheses to explain the discrepancies, and you go back and do more of these global and integrated experiments,” Hood says.

It’s a long way from modeling yeast to modeling a human being. But Hood believes that the knowledge gained from a model of a simple system can be scaled up. Such comparative genomics—the ability to learn about complex systems by modeling simpler systems that have similar genetics—is one of the most powerful tools in systems biology, Hood says. “The basic idea is that once evolution latches on to a good idea, it generally uses it over and over again,” he says. “But when you do these comparisons you have to be very aware that although the very basic strategy may be similar, there might be many elaborations.” So scientists must look for “control elements,” small elements such as binding sites for transcription factors that are conserved in both organisms.

The next step in modeling is to describe relationships mathematically. The Institute for Systems Biology has created a mathematical model of the relatively simple metabolic process by which yeast cells get energy by breaking down the sugar galactose. After defining all the genes in the yeast genome and the particular genes, proteins, and other small molecules that are known to play a role in the galactose metabolism pathway, the researchers created a model. Then they grew, in the presence of galactose and without, normal yeast as well as strains that had particular genes deleted. They analyzed the reactions using microarrays, quantitative proteomics, and databases of known physical interactions. “We can write down a series of differential equations, and we can choose parameters to put into those differential equations and can predict a lot of the behavior of the system,” Hood says.

Some scientists believe that current bioinformatics capabilities can handle many biological modeling tasks if a complex system is portrayed as a series of smaller models that overlap one another. But the experimental design must integrate all the “omics” from the beginning. “The phrase ‘data integration’ has been closely associated with buying big mainframes and having engineers design big databases,” says Eric Neumann, global head of knowledge management at Aventis Pharmaceuticals. All of this computational power is aimed at trying to combine information from experiments that don’t have an integrated design—for instance, trying to relate gene expression data from one study to protein data collected in a separate study.

Neumann says this integration can be done better for one-tenth of the cost by adopting good experimental design and focusing more on the downstream. Neumann’s ideal experiment involves collecting gene microarray data, protein levels, metabolite levels, clinical phenotypes, and serum biomarkers in one experiment. “If one compares across experiments, then unless everything is kept constant, the data may not be statistically equivalent,” he says. “Some high-level comparisons may be made if one accepts the independent interpretations from both studies, but these will often be more qualitative mergings than quantitative in nature.”

The integrated experiment design that Neumann favors would allow statistically valid merging, he says. That merging can be accomplished with several simple databases that all point to the same experimental design. The end result is a relational database that “looks like a big spiderweb,” Neumann says.

The ultimate object of systems biology is to understand how the elements and their interactions together give rise to the emergent properties of the system.

— Leroy Hood

John Doyle, a professor of control and dynamical systems at the California Institute of Technology, has likened the complexity of a biological system to that of a Boeing 777 jet. Each system needs only a small portion of its control systems for basic functioning (for instance, *Escherichia coli* can survive in the laboratory even when 90% of its genes are knocked out). The jet includes more than 100,000 components such as computers and sensors, most of which are not needed under ideal conditions, but which enable the plane to stabilize if conditions suddenly change. Likewise, biological organisms include complex control systems that kick in only during potential threats—such as variations in temperature or nutrients—to keep the organism stable.

In general, this complexity makes the organism robust. But some scientists hypothesize that such complexity can leave a system vulnerable to unplanned disruptions such as genetic mutation. The mutation may be tiny, but because the gene is involved in such a complex, multilayered control network, the tiny mutation can trigger a “cascading failure”—a kind of domino effect that leads to a major threat such as cancer or autoimmune disease. Mathematicians and engineers are at work on algorithms and other tools to better model this robust yet fragile nature and other aspects of complex biological systems.

## High Hopes for Tiny Tools

Some researchers say that developments in nanotechnology and microfluidics may revolutionize systems biology. Nanotechnology involves manipulating molecules smaller than 100 nanometers—the scale of viruses. Microfluidics, which is commonly used in ink-jet printing, uses pumps and valves to transport nanoliter volumes of fluids through microchannnels in a tiny glass or plastic chip. Hood says that nanotechnology and microfluidics will eventually enable scientists to make many different measurements in parallel and in small amounts of material. “In principle, you can make these measurements down to the single-molecule level,” he says.

In theory, researchers could create nanobiosensors no bigger than 100 nanometers that could be surgically implanted into the body or injected into the bloodstream to measure biomarkers of environmental exposure or diagnose problems in cell function. Suk says nanobiosensors could potentially measure processes as sensitive as the flux of calcium ions inside a cell.

David Walt, a professor of chemistry at Tufts University, is using fiber optic technology to develop tiny sensors that could be used to screen toxicants. Optical fibers are extremely fine strands of glass that can transmit light to and from a sample. Right now the sensors directly measure chemical changes in arrays of yeast or *E. coli* cells in response to toxicants such as mercury or the chemotherapeutic agent mitomycin C. The cells are fluorescently labeled, and the researchers monitor chemical changes by correlating those changes to changes in fluorescent intensity.

“The goal is to be able to measure solutions and determine their toxic potential,” Walt says. A similar method could eventually be used to quickly screen potential toxicants, replacing some animal testing. First, though, studies would need to be run to ensure that the cell-based arrays consistently yield the same results as animal testing, Walt says.

Suk is excited about the idea that nanobiosensors could enter cells and make direct measurements of their inner workings. “That will allow us to have a better understanding—maybe even a complete understanding for certain types of tissues—of how cells and systems communicate with each other,” he says. “If you can understand how a cell works, you can then scale up to tissues, then organs.” He predicts that nanobiosensors will make it much easier to measure human exposures in as soon as five years.

## A Surprising Challenge

Once all the measurements are made, what must happen to make the megamodels of systems biology a reality? Surprisingly perhaps, some of the players in the field say that the biggest challenge for systems biology isn’t technical—rather, it’s a matter of community.

One problem is the lack of a common language for systems biology. As with other multidisciplinary collaborations, all the players will need to develop a language they can share. That became apparent at a December 2003 retreat on systems biology that Suk organized for the NIEHS Division of Extramural Research and Training, where speakers included an engineer, a biochemist, a computer scientist, and a physician—each of whom approaches the field from his or her own perspective.

Most of us in this area now have educated ourselves. People end up learning what they need to learn to solve the problems they’re interested in solving.

— Charles DeLisi

Neumann agrees the biggest challenges for systems biology are human ones—language and data sharing. “There’s a side to it that involves data analysis by people who feel comfortable looking at data, writing programs, some numerics,” he says. But that analysis needs interpretation from scientists who know about disease and about environmental exposures.

Neumann believes that getting the relevant data to those who can interpret it is the biggest bottleneck for the field. “There are more papers than ever before in science, and most of us can’t read them all,” he says. Simple text queries such as those used to search literature databases capture words or phrases out of context. But it’s possible now, Neumann says, to populate scientific papers with embedded, machine-readable phrases that convey the relevance of the work. Such databases would use ontologies—formal, machine-readable definitions of terms and the relationships between those terms. Programs that know something about logic and relationships can help weed out irrelevant information, Neumann says, and help reveal connections between concepts.

In the 2004 *Proceedings of the Pacific Symposium on Biocomputing*, Daniel McShan of the University of Colorado and colleagues present an example of how such data mining can be used to predict the behavior of a biopathway. The team developed an algorithm to extract, or infer, biotransformation rules from the Kyoto Encyclopedia of Genes and Genomes (KEGG), a web-accessible database of pathways, genes, and gene expressions. Using KEGG, the team inferred 110 biotransformation rules about what happens when certain compounds interact. They used these rules as well as mathematical algorithms to predict how detoxification pathways would metabolize ethyl and furfuryl alcohol. The model’s prediction correlated with known patterns of alcohol metabolism.

Automated data mining tools are well on their way to development now. “Vendors are working on various applications that would support the enhanced linking of documents anywhere, and eighty to ninety percent of that [technology] already exists,” Neumann says. [For an example of one such tool, see “Literature Searchlight,” *EHP* 112:A872 (2004).]

The real test is how willing scientists are to use these tools. “We already take enough time footnoting our papers. Imagine if those footnotes were machine-readable,” Neumann says. Authors would use software that works like a spell-checker to screen their papers and choose the formal concepts that are most relevant. “So all of a sudden the whole search and review of literature changes overnight,” he says. “Right now we all are doing text mining and extraction by ourselves. But in the future it will be done by authors as they submit their papers.”

Neumann believes that PubMed Central and other efforts to make all federally funded research freely available will provide an opening for data mining to become commonplace. “To survive, the scientific publishers are going to have to ask, ‘What’s our added value?’ If added value can be putting [text] in a smart ontology—bingo. I think we will see quick embracement of this so that [scientific publishers] find a whole new market strategy,” he says.

DeLisi adds that more emphasis on computational and mathematical training for scientists will help make systems biology more mainstream. “Most of us in this area now have educated ourselves,” he says. “People end up learning what they need to learn to solve the problems they’re interested in solving.”

Several training programs in bioinformatics and systems biology exist, such as the one that DeLisi is involved in at Boston University and those at various University of California campuses. Other programs are under development. “I’m very optimistic,” DeLisi says. “Over the next ten to fifteen years I expect to see a very large shift toward a much more mathematical biology, and certainly toward a highly computationally intensive biology. I have no doubt that in the next ten to twenty years, biology will be the most computational of all the sciences.”

## Optimistic Predictions

Suk’s biggest hope for systems biology is that it will create more realistic models of complex environmental exposures. “We’re exposed to lots of chemicals but at very low concentrations over time,” he says. “We need tools to help us understand how complex exposures perturb complex systems.”

Such tools to help elucidate system interrelationships are forthcoming. For example, in the August 2004 toxicogenomics issue of *EHP*, Hiroyoshi Toyoshiba and colleagues at the NIEHS Laboratory of Computational Biology and Risk Analysis reported the creation of a statistical software program that quantitatively sorts gene expression data to identify which genes interact in a network. The team has used the program to determine the effect of the carcinogen tetrachlorodibenzo-*p*-dioxin on 11 genes in lung epithelial cells and the genes’ subsequent effect on the retinoic acid signal transduction pathway.

This program looks further than comparing a simple pair of genes. Instead it shows the relationships between a whole set of genes in a network. The study authors have said that when the tool is expanded to include other data such as protein levels, it will help researchers understand biopathways in cells, tissues, and eventually entire organisms.

David Schwartz, a professor of medicine and genetics at Duke University, acknowledges the benefit of the systems biology approach but tempers his enthusiasm with a focus on the here and now. “Systems biology may help us understand biological processes, but we have to put them into a context of human disease,” he says. Schwartz’s lab investigates how gene expression profiles can be used as preclinical markers to help understand the biology and genetics of complex environmentally related diseases such as asthma. “We can also use global ‘omic’ approaches to identify biologic pathways that are specifically affected by disease-based environmental toxicants,” he says.

But Suk is optimistic that systems biology will deliver on its promise for environmental health in the near future. “We’re only limited by two things—by our ability to [grasp] it and by money,” he says.

A working model of an entire biological system would possess enormous power for learning how environmental exposures result in disease. And some scientists say that many elements of such a model are within science’s grasp.

Systems biology may help us understand biological processes, but we have to put them into a context of human disease.

— David Schwartz

But fully embracing the systems approach will also require scientists to embrace change. They must create a new language for the field. They must design experiments always with the whole system in mind. They must even learn a new way of footnoting their papers. How will all this change happen? Like the intricate webs of systems biology models themselves, the answer is sure to be complex.

## Figures and Tables

**Figure:**